# A New Generation of Activated Carbon Adsorbent Microstructures

**DOI:** 10.1002/advs.202406551

**Published:** 2024-09-06

**Authors:** Ethan Grigor, Joseph Carver, Edric Bulan, Stuart Scott, YM John Chew, Semali Perera

**Affiliations:** ^1^ Department of Chemical Engineering University of Bath Bath BA2 7AY UK

**Keywords:** 3D Printing, Activated Carbon, Adsorption, Microstructures, Porous Materials

## Abstract

This work presents the successful manufacture and characterization of bespoke carbon adsorbent microstructures such as tessellated (TES) or serpentine spiral grooved (SSG) by using 3D direct light printing. This is the first time stereolithographic printing has been used to exert precise control over specific micromixer designs to quantify the impact of channel structure on the removal of *n*‐butane. Activated microstructures achieved nitrogen Brunauer Emmett Teller (BET) surface areas up to 1600 m^2^ g^−1^ while maintaining uniform channel geometries. When tested with 1000 ppm *n*‐butane at 1 L min^−1^, the microstructures exceeded the equilibrium loading of commercial carbon‐packed beds by over 40%. Dynamic adsorption breakthrough testing using a constant Reynolds number (*Re* 80) shows that complex micromixer designs surpassed simpler geometries, with the SSG geometry achieving a 41% longer breakthrough time. Shorter mass transfer zones were observed in all the complex geometries, suggesting superior kinetics and carbon structure utilization as a result of the micromixer‐based etched grooves and interlinked channels. Furthermore, pressure drop testing demonstrates that all microstructures had half the pressure drop of commercial carbon‐packed beds. This study shows the power of leveraging 3D printing to produce optimized microstructures, providing a glimpse into the future of high‐performance gas separation.

## Graphical abstract

This study compares the physical adsorption dynamics of 3D‐printed activated carbon (AC) microstructures with traditional AC structures. 3D printing allows for customizable geometric configurations, such as micromixers, to be incorporated into channel structures. The findings show that these microstructures offer greater breakthrough time and equilibrium capacity while reducing pressure drop by more than half compared to packed bed carbon.

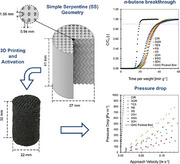

## 1 Introduction

The effective separation of target gases is a vital part of many industrial and energy generation processes, including carbon capture, biogas upgrading, and hydrogen purification.^[^ ^1^ ^]^ Most traditional gas separation technologies such as cryogenic distillation, membrane‐based, and absorption‐based separation are energy intensive.^[^ ^2^, ^3^ ^]^ Adsorption‐based separation is widely considered to be more energy efficient, low cost and can operate over a wide pH range,^[^ ^4^ ^]^ thus has the potential for intensifying separation processes. As demand for more efficient and environmentally sustainable gas separation procedures grows, developing adsorbents with tailor‐made structures and tunable surface properties becomes increasingly important.^[^ ^1^ ^]^

Activated carbon (AC) and metal–organic frameworks (MOFs) are commonly used for various gas separation, purification and catalysis applications, including volatile organic chemical (VOC) removal and natural gas desulphurization.^[^ ^2^, ^5^, ^6^, ^7^, ^8^, ^9^, ^10^ ^]^ MOFs are porous materials with high selectivity and versatility making them desirable for gas separation and catalysis, especially in the case of chemical adsorption;^[^ ^11^, ^12^, ^13^ ^]^ however, MOFs can exhibit poor thermal and chemical stability with issues of scalability. ACs are particularly attractive for gas separation due to their abundance, low cost, high surface area, chemical inertness, and stability.^[^ ^14^ ^]^ Extensive use of AC in industry has led to it being one of the most mature solid adsorbent technologies. Typically, AC precursors are coal and coconut derivatives due to their preferential pore size distribution and surface composition post‐activation for adsorption.^[^ ^15^ ^]^ However, several studies have demonstrated that biomass‐waste‐derived ACs can be utilized, and they yield comparable surface areas to the coconut and coal counterparts.^[^ ^16^, ^17^ ^]^

While ACs commonly exist in granulated form in packed bed applications, these processes suffer from channeling, pressure drop drawbacks and packing limitations, resulting in rapidly reduced adsorption performance.^[^ ^18^ ^]^ To overcome these limitations, activated carbon monolithic (ACM) adsorbents prepared using extrusion have gained considerable attention in gas separation systems. ACMs are structures composed of parallel channels with varying cross‐sectional shapes: typically square, circular, or hexagonal channels.^[^ ^19^ ^]^ Due to the thin channels (hundreds of microns) and low tortuosity flow paths, ACMs exhibit favorable mass transfer kinetics and offer significant pressure reductions. In carbon capture applications, ACMs have already been proven as having one of the highest gas permeabilities of structured carbons as well as low costs and pressure drops.^[^ ^20^ ^]^

By structuring adsorbent channels into tunable geometric configurations, ACMs have the potential to provide improved gas–solid contacts and enhanced adsorption/desorption properties compared to traditional granular activated carbons (GAC). However, the geometry of ACMs has been restricted to only straight‐through channels due to the limitations of traditional extrusion manufacturing techniques. These typically involve directly extruding carbon with a pre‐designed die.^[^ ^21^ ^]^ Consequentially, the ACMs have a potential risk of gas adsorptives passing through the structure without contacting the adsorbent surface (premature breakthrough). However, recent developments in 3D printing have realized the potential to produce almost any geometrical complex structure, from a range of materials. 3D printing offers layer‐by‐layer scale precision and functionality not achievable from extrusion construction methods. As a comparison to traditional extruded ACMs, 3D printing offers the potential to explore complex channel designs, such as serpentine and spiral channels, to improve adsorption dynamics by promoting mixing, flow turbulence, and extended flow paths.^[^ ^22^ ^]^

One of the main 3D printing techniques is photopolymerization, which refers to the curing of photopolymer resin with a laser, UV, or LED light source.^[^ ^23^ ^]^ Among photopolymerization techniques, stereolithography (SLA) is one of the most promising for the fabrication of AC structures. In SLA, a UV laser is used to trace and cure the model's cross‐section, forming thermosets from photocured commercial resins with high resolution.^[^ ^24^ ^]^ A more recent technique based on SLA uses Direct Light Projection (DLP) to expose an entire layer of resin at once.^[^ ^25^ ^]^ In this technique, the cured layer is formed on a build plate, which lifts out of the resin vat. The build plate then moves back down to a layer thickness above the bottom of the resin vat, and the next layer is subsequently cured. Competitive technological breakthroughs in photopolymerization have reduced print times by 25–100 times whilst also retaining feature resolution below 100 micrometers.^[^ ^23^ ^]^ As a result, there has been a rapid uptake in its use in various fields of research and application, including water treatment,^[^ ^26^, ^27^ ^]^ electrochemistry,^[^ ^28^ ^]^ and biomedical applications.^[^ ^29^ ^]^

There is limited research performed into 3D printing microstructures with incorporated micromixer designs. Work done by Steldinger et al.^[^ ^24^ ^]^ demonstrated that SLA could be used to print a simple cell network adjoining eight tetragonal cubic centered unit cells, with a diameter of 5.7 mm each, to form a single monolithic structure, which could then be thermally treated to form an AC monolith. Further, a study performed by Zafanelli et al.^[^ ^30^ ^]^ identified that these activated carbon monolithic structures could potentially be used for post‐combustion CO_2_ capture. The ACMs formed had BET surface area's varying from 800 − 1000 m^2^ g^−1^, with an open structure design—that is to say, the channels protruded into the outer walls of the structure. More recent work by the current authors^[^ ^22^ ^]^ identified that using SLA, more complex micro‐sized internal geometries could be printed to benefit gas adsorption. These geometries were constructed using a wall thickness of 1.12 mm and a channel diameter of 1.68 mm. This paper explored the potential of utilizing spiral, chevron and stepped channel designs to induce turbulence within the internal structure of the microstructures. The research demonstrated the feasibility for implementation of complex channel designs to be printed into the microstructures to further develop mixing in order to enhance mass transfer for adsorption.

This study aims to create adsorbent microstructures that exhibit improved breakthrough times, equilibrium loadings and reduced pressure drops in comparison to traditional GAC‐packed beds and extruded ACM structures, utilizing state‐of‐the‐art DLP printing. Until now, 3D printing of AC microstructures has only been explored with simple geometries,^[^ ^22^, ^24^, ^31^ ^]^ and to the author's knowledge, no studies have been published regarding the fabrication and activation of 3D printed micromixer designs. This study focuses on developing complex channel geometries in adsorbent microstructures based on research on effective micromixers in microfluidics. This represents a step‐change contribution to the area of 3D printed adsorbent microstructures, which until now has mostly been limited to using direct ink writing technique to extrude structures from zeolite pastes, metal–organic frameworks, and carbonaceous materials.

## 2 Design of 3D Printed Adsorbent Microstructures

**Figure 1:**
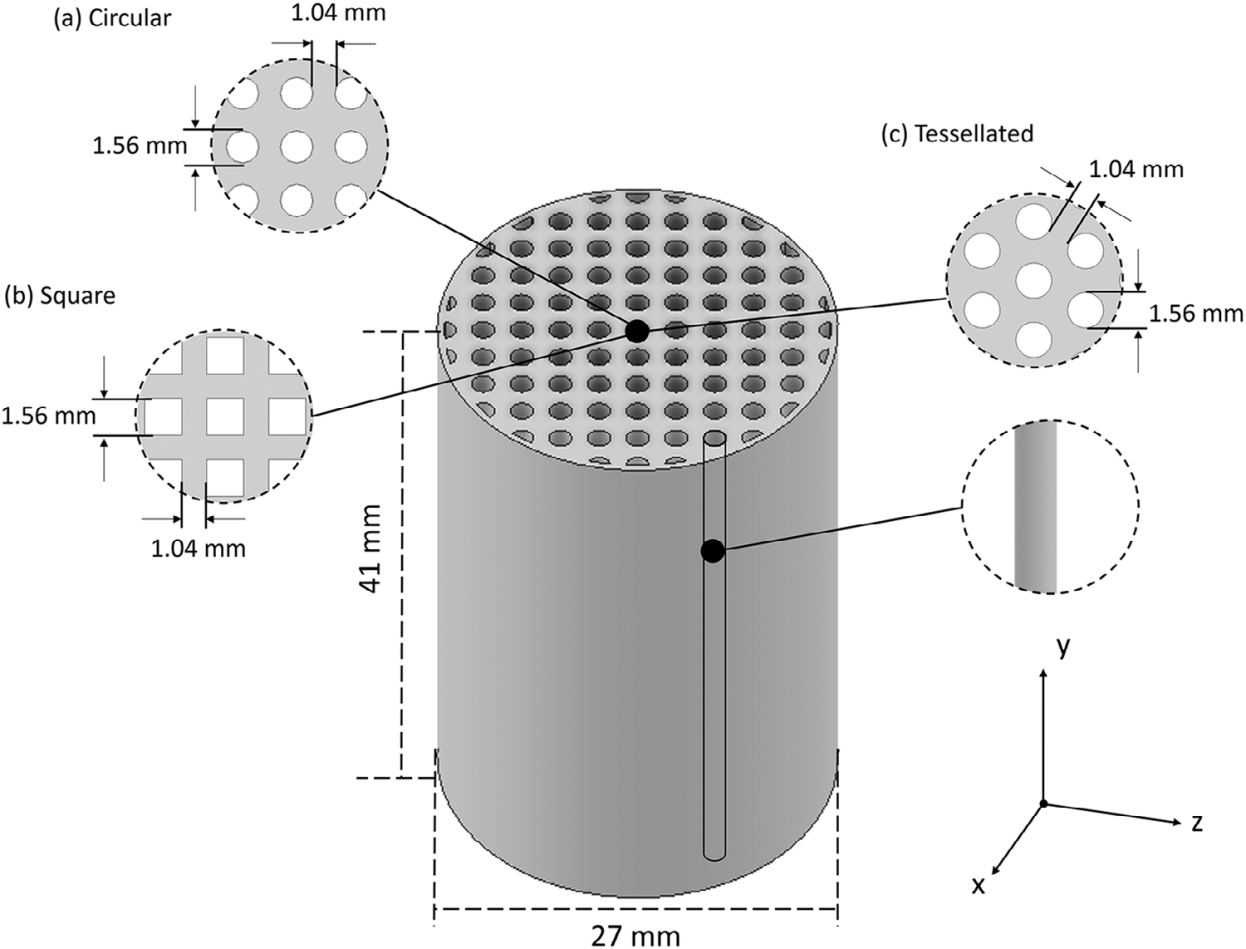
CAD designs for the simple geometry adsorbent microstructures: a) circular, b) square and c) tessellated channel designs.

Three simple geometries, namely, Circular (CIR), Square (SQR), and Tessellated (TES) straight‐through channels are presented in **Figure** **1**a–c respectively. Comparable to straight‐through channels found in extruded ACMs, these simple geometries provide a benchmark to assess the complex geometries developed. These adsorbent microstructures were designed in Autodesk Inventor with dimensions based on previous work by the current authors.^[^ ^22^ ^]^

These structures were designed with parameters height, *H*, diameter, *D*, and equivalent inlet/outlet channel diameter, *D*_CH_, 41, 27, and 1.56 mm, respectively. The distance between each channel center point was 2.6 mm for all simple structures. Adjusting the circular design from a grid to a honeycomb orientation, referred to as tessellated (Figure 1c), yields an increased cells per square inch (CPI) in the activated sample, from 201 to 223, whereas, in the square structure, this was found to be 201.

**Figure 2:**
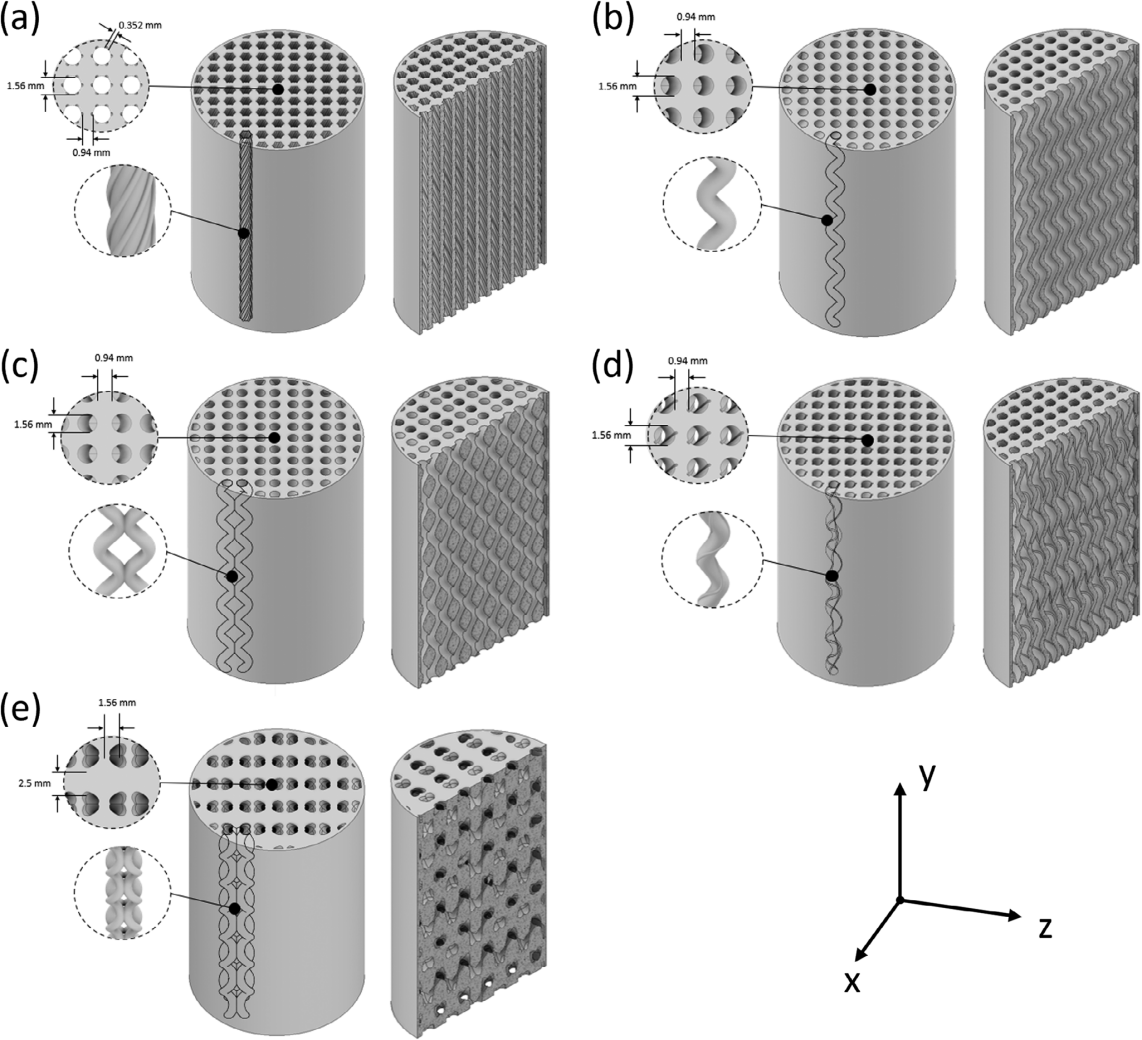
CAD designs for the complex geometry adsorbent microstructures: CAD designs of complex geometry adsorbent microstructures: a) Rifled Spiral, b) Simple Serpentine, c) 2D Helix, d) Serpentine Spiral Groove, and e) 3D Helix channel designs.

The complex geometries, Rifled Spiral (RS), Simple Serpentine (SS), 2D Helix (2DH), Serpentine Spiral Grooved (SSG), and the 3D Helix (3DH), as seen in **Figure** **2**a–e, respectively, have been developed based on numerical studies conducted on mixing efficiencies and pressure drop in 3D micromixers available in the literature.^[^ ^32^, ^33^, ^34^, ^35^, ^36^ ^]^ Studies by Mondal et al.,^[^ ^37^ ^]^ Wang et al.,^[^ ^32^ ^]^ and Afzal et al.^[^ ^36^ ^]^ highlighted the applications of micromixer designs in the area of microfluidics for chemical, biological, and biochemical synthesis and analysis. These applications are all geared toward numerical analysis and liquid interactions, as opposed to gas interactions, considered in this work.

**Rifled Spiral** Figure 2a: RS comprises etched grooves patterned to spiral axially along a circular straight‐through channel. The introduction of grooves has been investigated in micromixers numerically by Mondal et al.^[^ ^37^ ^]^ and Wang et al.,^[^ ^34^ ^]^ with further work by Stroock et al.^[^ ^38^ ^]^ demonstrating the advantages of grooves along the channels to generate secondary flows with a negligible resistance to the fluid flow. To implement a single rifle on the channel, a semi‐circle was added to the channel edge and set to coil three revolutions around the channel for a length, L, of 41 mm. This process was repeated for a total of six rifles evenly distributed on a single channel, yielding a CPI of up to 240 in activated samples (see Section S1, Supporting Information).

**Simple Serpentine** Figure 2b: SS was developed to generate chaotic advection effects. The serpentine channels were constructed by sweeping a compressed sine curve (to limit the radial disruption to adjacent channels) axially for a length equal to 41 mm, according to Equation 1. Incorporation of a sine curve elongates the true path length compared to straight‐through channels while maintaining a CPI of up to 213 in activated samples (see Section S1, Supporting Information).

(1) f(y)=0.8siny

**2D Helix** Figure 2c: 2DH has similar benefits to that of the serpentine with chaotic advection effects; however, additional mixing arising from interconnected multichannel networks, as seen in recent microfluidic work.^[^ ^39^, ^40^, ^41^ ^]^ The helical structure was developed using alternating out of phase by 90° serpentine channels, developed by compressed sine and cosine curves, defined by Equations (1) and (2). These functions were swept axially down the length of the microstructure (41 mm), arising in an extended true path length with interconnectivity for enhanced mixing.

(2) $$g\left(y\right)=0.8\cos y+\frac{\pi }{2}$$

**Serpentine Spiral Groove** Figure 2d: SSG is a combination of simple serpentine and rifled spiral channel designs. Developed to capture the benefit utilizing both groves and chaotic advection mixing strategies this design is comparable to the grooved spiral micromixer studied by Rafeie et al.^[^ ^42^ ^]^

**3D Helix** Figure 2e: 3DH was developed, building upon the 2D Helix design and comprises of spirals in alternating directions. This means that each spiral channel intersects with four alternate and adjacent spiral channels resulting in high interconnectivity between the channels. This design was developed based on work by Liu et al.^[^ ^41^ ^]^ on a cross‐linked dual helical micromixer. These spirals are defined by the parametric Equations (3)–(5).

(3) x(t)=±0.8cost

(4) y(t)=t

(5) z(t)=±0.8sint

A summary of the simple and complex geometries dimensions can be found in Section S1 (Supporting Information).

## 3 Results and Discussion

### 3.1 Physical Characterization

**Figure 3:**
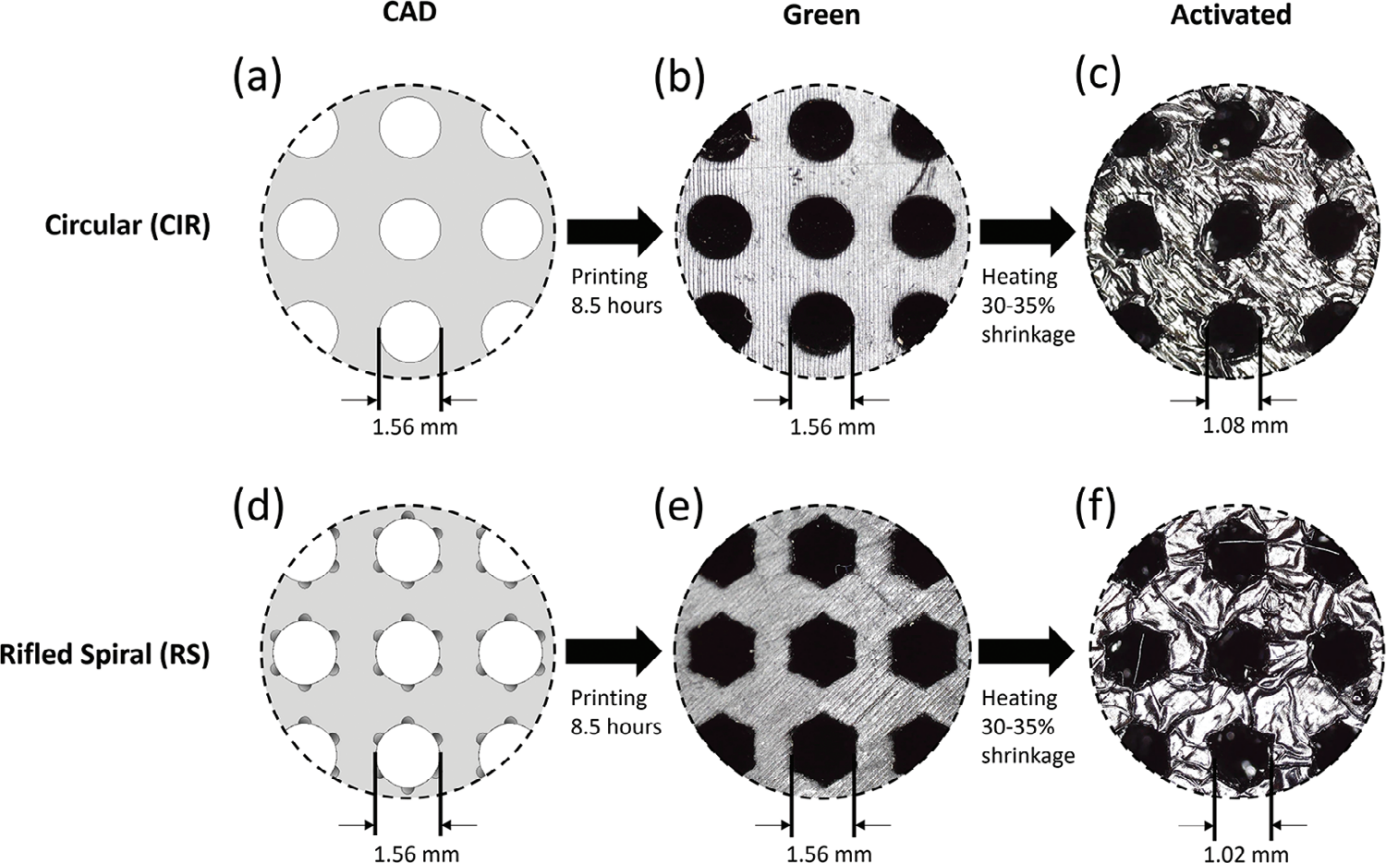
CAD drawings (a,d) and microscopic images (b,c,e,f) of CIR (a–c) and RS (d–f) activated carbon microstructures.

**Figure** **3** shows the CAD models and microscopic images of the adsorbent microstructures at three stages during their preparation. Images of the green, Figure 3b,e, and activated adsorbent microstructures, Figure 3c,f, show a strong adherence to the initial CAD model. In Figure 3f of the RS channel design, it is evident that fine details of the grooves in the final activated structure are poorly defined compared to the original CAD model. The cracking seen in Figure 3c,f is a product of the oxidative stabilization, carbonization and activation procedures. carbonization removes carbon heteroatoms from the structure which causes this cracking. Activation widens existing pores while also “opening” up closed porosity within the structure. These cracks create a more open pore network, increasing the accessibility of the microporosity within the structure, while not impacting the integrity of the structure.

Following activation, the adsorbent microstructures experienced a degree of activation alongside an overall volume decrease of ≈35–40%. The degree of activation was defined as the mass of carbon remaining after activation, see section S.1 for further detail. In co‐occurrence with the volume decreasing, the microstructures experienced a uniform shrinkage in axial and radial directions; however, the original aspect ratio designed in the CAD drawings was maintained in the activated microstructure. The majority of this mass loss can be attributed to the polyacrylate base of the resin. During carbonization, heteroatoms (non‐carbon atoms) were expelled from the structure, resulting in a porous carbon‐rich product. The activated microstructures were characterized by the degree of activation. Typically, a 35–45% degree of activation was achieved across the AC microstructures as targeted.

**Figure 4:**
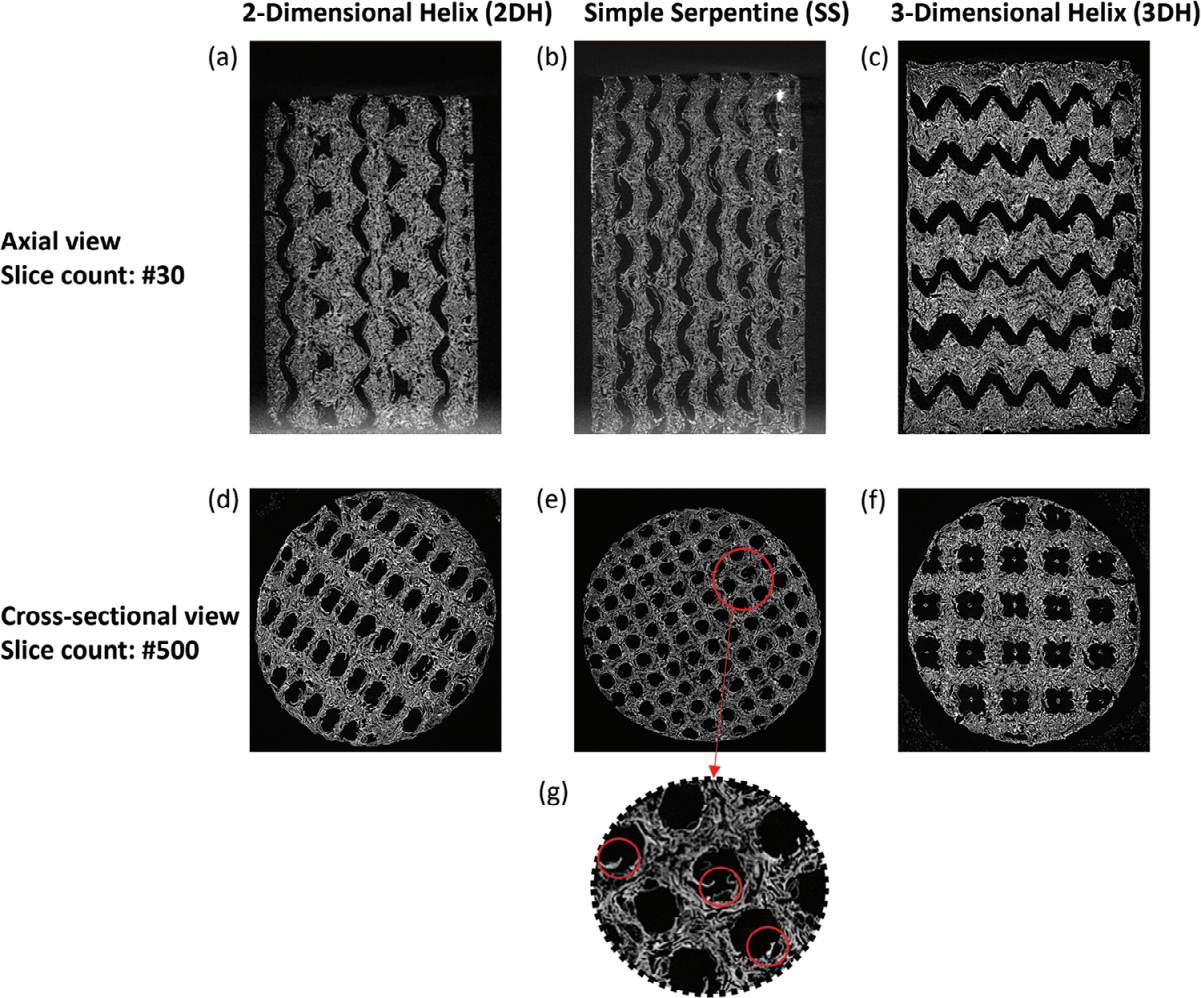
CT scans of the axial view (a–c) at slice count 30 and cross‐sectional view (d–f) at slice count 500 for the 2DH (a,d), SS (b,e) and 3DH (c,f) complex geometries, (g) illustrates channel flaking.

**Figure** **4**a–f shows axial and face views of the CT scans taken of the 2DH, SS and 3DH complex geometries. From Figure 4a–c it was observed that the channels had formed as designed with clear definition in the axial direction, and in the case of the 3DH, axial and radial direction. The cross‐sectional views, Figure 4d–f also demonstrated clear well‐defined channel structures. Although, Figure 4e has some minor channel blockage occurring as a result of deformation from the heating protocol. This flaking effect did not impede *n*‐butane breakthrough testing but did have an impact on the pressure drop testing due to extra resistances exerted from disturbances in flow patterns.

**Figure 5:**
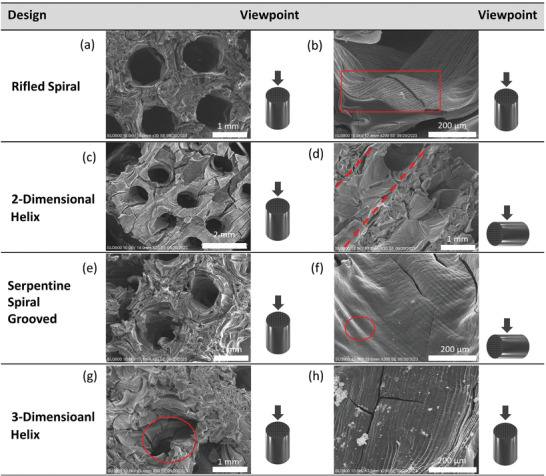
SEM images and from top (a,b,c,e,g,h) and side views (d,f) for the RS: (a,b), 2DH: (c,d), SSG: (e,f) and 3DH: (g,h) channel designs.

SEM micrographs shown in **Figure** **5**a,c,e,g demonstrated that the channels on the face of the microstructure were well defined and after activation maintained resemblance to the original CAD designs. The additional cracks forming resulted in additional porosity for adsorption and did not impact the mechanical integrity of the structures. Figure 5d,g shows the interlinking nature of the 2DH and 3DH geometries had formed correctly and withstood activation, such that no obstruction nor deformation of the geometries took place. However, in Figure 5a,e, for the rifled aspect of the designs, the grooves of the channels were less distinct which was attributed to limitation with the resolution of the 3D printer used. In Figure 5b,f,h print layers were observed alongside minor debris and cracking. The debris was not severe enough to impede channel formation nor pore structure (see section s.4 Supporting Information for SEM micrographs of simple channel geometries).

### 3.2 Mechanical Performance

**Figure 6:**
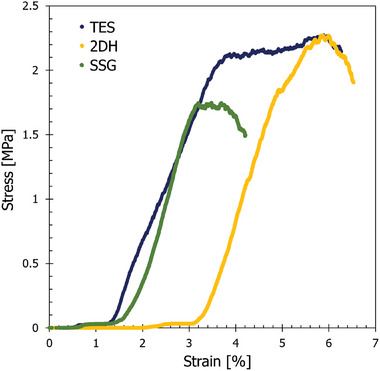
Compressive strength of 3D printed activated carbon microstructures TES, SSG, and 2DH.

The stress–strain curves obtained from compressive strength testing are depicted in **Figure** **6**. Based on these curves, it was determined that the yield strengths of TES, SSG, and 2DH were 2.03, 1.61, and 1.84 MPa, respectively. Given that the TES structure features uniform “straight‐through” channels without complexity, it was anticipated to exhibit a higher yield strength. On the other hand, the 2DH and SSG geometries contain “hollow” points due to the sine curvature of the channel, which may have compromised the structural integrity under compression load.

Literature sources on ACMs have reported varying yield strengths ranging from 0.0107 to 24 MPa,^[^ ^43^, ^44^, ^45^ ^]^ depending on factors such as precursor materials, processing conditions, and activation conditions. In a recent study by Rangel‐Sequenda et al. (2022),^[^ ^46^ ^]^ the yield strengths of 3D printed ACMs ranged from 1 to 3.5 MPa, aligning closely with the values observed in this study. Moreover, the fracture regions (breaking point of the material under stress) of the microstructures seen in Figure 6 were 6%, 3%, and 6% for TES, SSG, and 2DH, respectively. These values align with the range reported in the literature, which typically ranges from 4.5% to 6%.^[^ ^43^, ^44^, ^45^, ^46^ ^]^

### 3.3 Nitrogen Isotherms

The nitrogen isotherms for the simple and complex adsorbent microstructure channel designs exhibit a type I shape, according to the IUPAC classification, characteristic of microporous materials (see Section S.5 for isotherms, Supporting Information).

**Table** **1** summarizes the BET surface area, S_*BET*_, the pore width and degree of activation for all samples. The S_*BET*_ measured by nitrogen adsorption shows an increase with degree of activation, from 626 m^2^ g^−1^ for the Circular sample with a 28.4% degree of activation to 1605 m^2^ g^−1^ for the Tessellated sample with a 41.9% degree of activation. This is a result of most of the micropore volume being created during activation, which modifies textural properties such as surface area, micropore volume, and pore size distribution. By comparing pore volumes and pore size distributions calculated for the different geometries, it was observed that pore volumes for degrees of activation less than 35% was significantly lower than those with higher degrees of activation. This suggests that the microporous network either has not been exposed after activation (insufficient activation), or the pores that have been exposed were to narrow such that they were inaccessible to nitrogen, meaning they would be ineffective at *n*‐butane adsorption. Though, it must be considered that the geometry of the microstructures would affect the activation procedure due to the tortuous path the carbon dioxide would have to travel throughout the microstructure. For instance, the TES structure has one of the larger CPI's, 223, when compared to other geometries, 83–218, therefore it would be expected for more carbon dioxide to be exposed to more of the microstructure, leading to an increased degree of activation. This implied CPI had a correlation to the degree of activation.

**Table 1:** N_2_ physisorption data for 3D printed adsorbent microstructures and GAC.

	Channel Designs	Degree of Activation [%]	BET Surface Area [m^2^ g^−1^]	Pore Width Range [nm]	Pore Volume [cm^3^ g^−1^]
Simple	Circular (CIR)	28	630	0.55–2.00	0.23
	Square (SQR)	27	680	0.55–2.00	0.25
	Tessellated (TES)	42	1600	0.55–3.32	0.65
Complex	Rifled Spiral (RS)	37	950	0.55–1.61	0.35
	Simple Serpentine (SS)	40	1200	0.57–4.22	0.53
	2D Helix (2DH)	22	810	0.57–1.48	0.29
	Serpentine Spiral Groove (SSG)	33	930	0.57–2.80	0.35
	3D Helix (3DH)	38	1100	0.55–3.55	0.47
	Granular Activated Carbon (GAC)	—	940	0.590	0.46

### 3.4 Dynamic Adsorption

**Figure 7:**
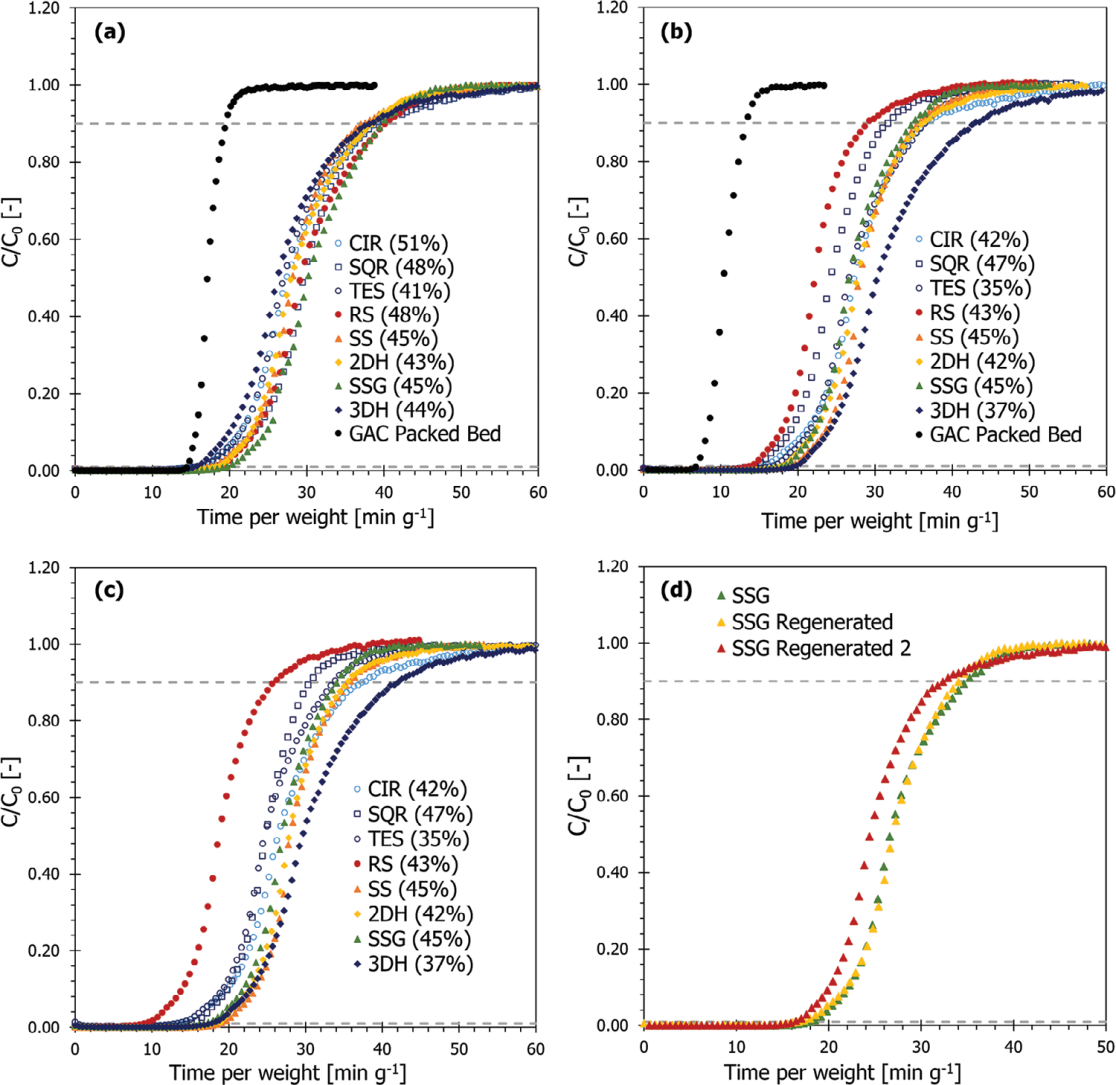
Normalized *n*‐butane breakthrough curves for AC microstructures and GAC at a) 1 L min^−1^. b) *Re* = 83, c) regenerated samples presented in (b) tested at *Re* = 83 and d) multiple regeneration cycles of SSG at *Re* = 83. C_0_ = 1000 ppm *n*‐butane. The legend describes the type of structure (CIR = Circular) and the corresponding degree of activation in parentheses.

The breakthrough properties of the adsorbent microstructures were evaluated at 25°C, atmospheric pressure and at constant flow rate, 1 L min^−1^ (**Table** **2**) and also at constant *Re* = 83 (**Table** **3**). It was acknowledged that the highest Reynolds number assessed was 103, in the case of the 3DH sample at 1 L min^−1^, meaning that all flow regimes tested were laminar.^[^ ^47^ ^]^ The 1000 ppm *n*‐butane breakthrough profiles corresponding to these conditions are presented in Figure 7.

Three performance indicators have been established and applied in this study: breakthrough time, *t_*b*_*, equilibrium loading, *q_*e*_* and mass transfer zone (MTZ) length (see Section S3, Supporting Information). The breakthrough time is defined as the time by which the effluent gas concentration reaches 1% of the initial feed gas concentration (10 ppm *n*‐butane), which is typical for industry standards. To facilitate direct comparison, the breakthrough time has been normalized relative to the mass of adsorbent. The equilibrium loading is characterized as the ratio of the mass of *n*‐butane adsorbed to the mass of adsorbent. The length of the MTZ is calculated as the duration between t_*b*_ and the time at which 90% saturation is achieved. These performance indicators were calculated from the *n*‐butane breakthrough curves.

As summarized in Table 2, all simple and complex adsorbent microstructure designs surpassed the GAC‐packed bed in terms of breakthrough time and equilibrium loading. However, it was acknowledged that the GAC was an impregnated sample and intended to tackle a wider range of chemicals. As such, the equilibrium loading and breakthrough time would have been slightly reduced from this. As a result, its capacity for physisorbed chemicals was compromised accordingly. In the case of the SSG geometry (best performing microstructure), a 28% increase in breakthrough time and 60% increase in equilibrium loading was demonstrated. The simpler straight‐through channel geometries such as CIR and SQR, had similar breakthrough times when compared with the UFR GAC, but higher equilibrium loadings. Although, when assessing **Figure** **7**a–c it was evident that the nature of the breakthrough curves were distinctly different. The GAC, in all experiments, had a more efficient bed utilization (as seen by the steeper gradient and shorter MTZ – see Table 2). This shorter MTZ is characteristic of GAC and demonstrated the benefits of finer particle size for diffusion and hence better internal and external mass transfer. The 2DH structure had the shortest MTZ length from the microstructure and was longer than the GAC by ≈64% (Table 2). This implied the diffusion kinetics within the microstructures were slower than that of the packed bed. Although a longer MTZ length was demonstrated, higher equilibrium loading, exhibited by the adsorbent microstructures with enhanced breakthrough times, support the strong potential for resin‐based 3D‐printed adsorbent microstructures as effective materials for gas separation processes.

**Table 2:** Summary of channel designs with their associated breakthrough time, loading, MTZ length determined from 1000 ppm *n*‐butane breakthrough results assessed at 1 L min^−1^ flow rate alongside their degree of activation.

	Channel Designs	Degree of Activation [%]	Breakthrough Time, t_*b*_ [min g^−1^]	Equilibrium Loading, q_*e*_ [g g^−1^]	MTZ Length [mm]
Simple	Circular (CIR)	51	15.5	0.082	16.7
	Square (SQR)	48	15.5	0.079	17.9
	Tessellated (TES)	41	16.1	0.084	16.4
Complex	Rifled Spiral (RS)	37	17.7	0.078	16.1
	Simple Serpentine (SS)	45	17.1	0.072	16.0
	2D Helix (2DH)	43	17.9	0.084	14.1
	Serpentine Spiral Groove (SSG)	45	19.4	0.080	15.3
	3D Helix (3DH)	44	15.4	0.074	17.1
	Granular Activated Carbon (GAC)	—	15.1	0.050	8.6

Reproducibility of the AC microstructures was consistent. All prints formed as intended and after activation still maintained mechanical integrity and well‐defined channel geometries. This reproducibility can be observed in Section S6 (Supporting Information). The expected error for a breakthrough time was ≈5%, equilibrium loading 2% and MTZ length 7%.

When assessing Table 2, it could be attributed to that a lower degree of activation would imply a lower breakthrough time or equilibrium loading. However, this trend has not been observed. The lowest degree of activation microstructure, RS, had a comparable breakthrough time, 16.11 min g^−1^, when compared to the most activated microstructure, CIR, 15.54 min g^−1^, despite the difference in activation being in excess of 13%. From this, it can be inferred that the geometries themselves were extending the breakthrough times and equilibrium loadings, which was resultant from the induced turbulence and mixing effects caused by the complex geometries.

As GAC was compared as a reference material in these experiments, the comparison of simple to complex geometries was another benchmarking standard. Table 2 shows all the complex geometries had longer breakthrough times and maintained narrower MTZ lengths, excluding the 3DH which appeared to have a similar MTZ length than some of the simpler geometries (0.47 and 0.76 mm for CIR and TES respectively). This was a product of the continuous interlinking nature of the 3DH and extending the true path length of the internal channels by ≈30% compared to CIR, SQR, and TES. Yet, the increased path length from SS, SSG, and 2DH, ≈15% compared to the simple geometries, yielded shorter MTZ lengths by up to 26% (SQR compared to 2DH). Also, increased cell density benefits longer breakthrough times, being demonstrated by TES versus CIR and SQR, with CPIs 223, 201, and 201 respectively. This indicates that the TES geometry was an ideal benchmark geometry to compare the complex geometries to, as these geometries contained CPIs ranging from 83 to 218. Table 2 and Figure 7a support that incorporation of spirals and serpentine channels increases true path length and enables gas‐adsorbent contact to occur over a greater distance. Further, it was theorized that the complex geometries increased the residence time of the *n*‐butane within the structure. This prolonged exposure increased the potential accessibility of the adsorbent sites to the gas, extending breakthrough time and MTZ length. This effect was amplified by the cracks that formed during activation.

At a constant flowrate of 1 L min^−1^ the SSG and 2DH geometries had the longest breakthrough times, 19.4 and 17.9 min g^−1^ respectively, while maintaining the shortest MTZ lengths, 15.27 and 14.14 mm respectively. The geometries increased breakthrough time by over 10% compared to simple geometries and decreasing the MTZ length by over 10% also.

Figure 7a shows that at 1 L min^−1^ it appeared that the physical adsorption kinetics of the microstructures were similar, as the shape of the curves followed a similar pattern. At constant flowrate 1 L min^−1^, visually inspecting the curves to distinguish between different geometries was not effective due to their clustered nature. A method utilized to differentiate between these geometries was testing at *Re* = 83. Doing so generated breakthrough curves that clearly demonstrate the impact of different geometries, as shown in Figure 7b and Table 3.

Figure 7b distinctly shows the difference between the microstructures for *n*‐butane adsorption at constant Re. In order to maintain constant Re, the flowrates of *n*‐butane were adjusted between 0.80 and 1.68 L min^−1^ (Table 3). Thereafter, the breakthrough time, which is a function of the flowrate of *n*‐butane, and thus may not be a suitable indicator of performance. Higher through‐puts of *n*‐butane will lead to earlier breakthrough times, as for any material. Therefore, it is proposed that equilibrium loading and MTZ length would serve as more reliable performance indicators. However, CIR which had the lowest flowrate of 0.96 L min^−1^, had a corresponding breakthrough time of 15.17 min g^−1^. This breakthrough time was over 20% shorter when compared to the SS, 2DH and SSG geometries which had higher flowrates varying from 1.07 to 1.08 L min^−1^ (as shown in Table 3). This demonstrated that in spite of the increased flowrate experienced by these complex geometries, the interlinking nature and extended tortuous path led to a superior uptake of *n*‐butane and thus increased breakthrough time.

**Table 3:** Summary of channel designs with their associated breakthrough time, loading, MTZ length determined from 1000 ppm *n*‐butane breakthrough results assessed at constant Reynolds number (Re  83) alongside their degree of activation.

	Channel Designs	Flow Rate [L min^−1^]	Degree of Activation [%]	Breakthrough Time, t_*b*_ [min g^−1^]	Equilibrium Loading, q_*e*_ [g g^−1^]	MTZ length [mm]
Simple	Circular (CIR)	0.96	42	15.2	0.071	17.4
	Square (SQR)	1.22	47	14.9	0.076	15.0
	Tessellated (TES)	1.10	42	16.6	0.089	16.7
Complex	Rifled Spiral (RS)	1.31	43	13.6	0.079	15.9
	Simple Serpentine (SS)	1.07	45	18.7	0.083	14.0
	2D Helix (2DH)	1.08	42	17.7	0.082	15.0
	Serpentine Spiral Groove (SSG)	1.08	45	17.9	0.079	14.4
	3D Helix (3DH)	0.80	37	21.4	0.070	15.6
	Granular Activated Carbon (GAC)	1.68	—	7.3	0.049	12.6

In terms of equilibrium loading, enhanced uptake was observed in the complex designs. Among the complex geometries, the 2DH design consistently demonstrated the highest equilibrium loading across the range of activation degree tested (0.082–0.084 g g^−1^—see Tables 2 and 3). As discussed, this can be attributed to the serpentine channels, which increase the channel length, thereby facilitating diffusion over a greater length (exposing the *n*‐butane to a greater surface area of carbon). Furthermore, the interlinking channels enable the fluid path to be multi‐directional, promoting continuous mixing and allowing *n*‐butane to access activated carbon in other regions of the adsorbent microstructure. However, the uptake for the 3DH design was comparably lower, an equilibrium loading of 0.070 g g^−1^ (Table 3), to all other complex structures. This is suggested to be due to the lower surface area of activated carbon within the channels as a result of the multiple repeating crossing regions which reduces the exposure of *n*‐butane to activated carbon despite the increased mixing.

Upon further analysis of Table 3, two comparable complex geometries, SS and SSG, were observed to exhibit similar flowrates (1.07 and 1.08 L min^−1^, respectively) and a degree of activation within 0.1% of each other. Interestingly, the SSG geometry, while having only a slightly longer MTZ length (0.41 mm difference), showed a lower equilibrium loading by 0.004 g g^−1^ compared to SS. Objectively, these differences were minor, and indicative that as suggested previously, the grooves in the SSG had not formed as intended; hence, did not provide any additional benefit over the SS geometry. This was also observed in Figure 7b, where the *n*‐butane breakthrough curves demonstrated a similar profile with negligible differences.

Figure 7c shows the microstructures being tested at a constant Reynolds (83), after regeneration under a vacuum. Regeneration and reuse are typically used in industry for solid adsorbents in large scale separation processes, such as temperature swing adsorption. The microstructures maintained consistent physical adsorption dynamics to the original sample, with some cases demonstrating improved *n*‐butane uptake and shorter MTZ lengths. Curve shape also remains consistent when compared to Figure 7b, demonstrating the capacity for these structures to be regenerated and maintain consistent performance (also seen in Section S.6, Supporting Information). The increased performance in some cases could be attributed to the thermal cycling effect, desorbing the *n*‐butane adhered to the adsorbent and increasing the degree of activation and stability of the microstructure. This is further evidenced in Figure 7d, where the SSG sample has been regenerated for two cycles.

The second regeneration cycle of SSG seen in Figure 7d had breakthrough time, equilibrium loading and mass transfer zone length equal to 16.24 min g^−1^, 0.075 g g^−1^, and 14.8 mm respectively. Between the “fresh” sample (not yet used for breakthrough) and the first regeneration cycle, the breakthrough time reduced by 1.2 min g^−1^, a slight increase in equilibrium capacity by 0.001 g g^−1^ and a lengthening of the MTZ by 0.74 mm. These changes are minimal when compared with original performance as evidenced by the nature of the curve seen in Figure 7d. Moreover, the second regeneration cycle only reduced the breakthrough time by a further 0.5 min g^−1^, suggesting the cycling stability of the microstructure was strong as the performance standard from fresh to two regeneration cycles was within 10%.

### 3.5 Pressure Drop

**Figure 8:**
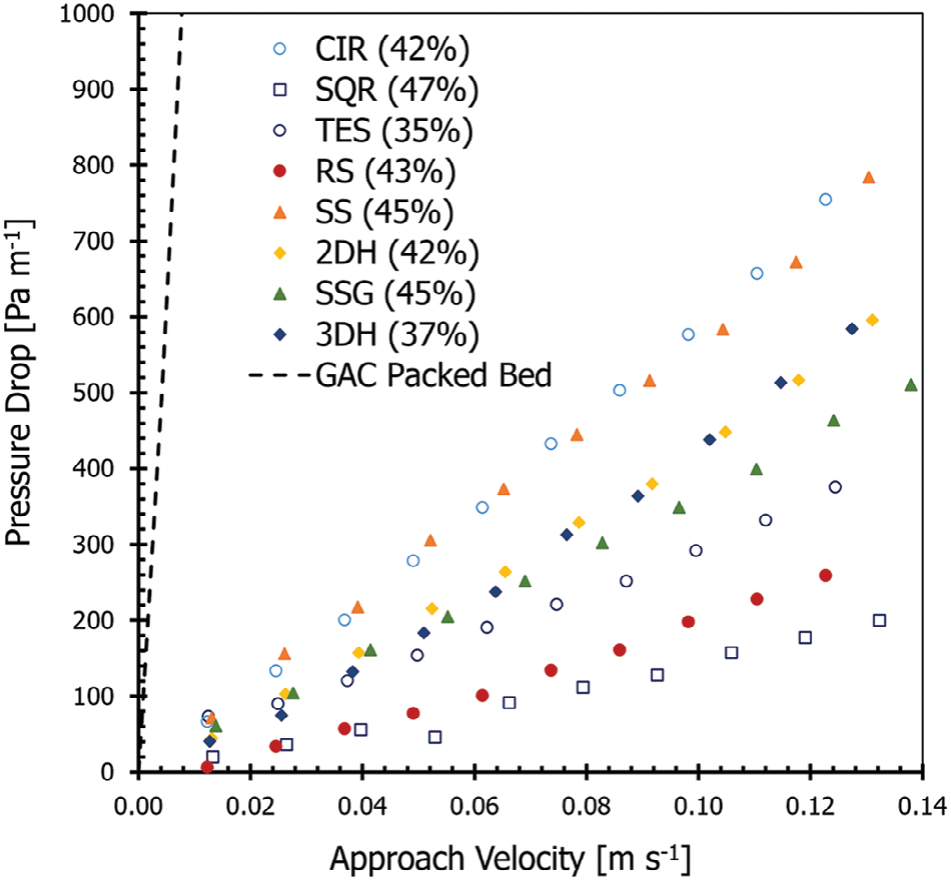
Experimental pressure drop for AC microstructures and GAC packed bed. The legend describes the type of structure (CIR ‐ Circular) and the corresponding degree of activation in parentheses.

Pressure drop is an important parameter due to its impact on system performance such as energy losses. The pressure drop results obtained for the adsorbent microstructures and an equivalent GAC‐packed bed are presented in **Figure** **8**. Figure 8 shows the GAC‐packed bed exhibited the highest pressure gradient, while the simple straight‐through adsorbent microstructures exhibited the lowest. The high‐pressure drop in the packed bed was attributed to the higher average velocity which led to higher inertia losses. The pressure loss for the simple and complex microstructures accounts for less than 5% of the pressure drop of the packed bed, comparable to results from Scott et al. (2023).^[^ ^22^ ^]^

When interpreting the pressure drop results for the microstructures it must be considered that additional cracking, as shown in Figure 4 and Figure 5, contribute to surface roughness increasing the overall pressure drop experienced. Furthermore, as seen in Figure 4e, some potential channel blockage can occur which would also distort flow creating larger pressure gradients within the system. Among the adsorbent microstructures, the TES and RS adsorbent microstructure designs performed with the lowest pressure drop. These are straight‐through channels with the lowest tortuosity with the highest channel density. The impact of reducing the channel density can be assessed by comparing the pressure drop between the TES to the CIR design, which approximately doubles the pressure drop experienced.

As expected, increasing the complexity of the channel geometries in the adsorbent microstructure designs (RS, SS, 2DH, SSG, and 3DH) resulted in increased pressure drops. The SS channel design had a 40% increase in the pressure drop compared to the 3DH and 2DH channel designs despite a relatively lower tortuosity. This was suggested to be due to cracking and flaking within the channels of this design resulting in increased surface roughness. As it would be expected that structures containing serpentine geometries in addition to multichannel interlinking would impose greater pressure drops than those with just serpentine structures.

### 3.6 Comparison of Adsorbent Microstructures Performance

By introducing complex geometries into the channels of the adsorbent microstructures, it has been demonstrated that improved external mass transfer into the carbon walls can be achieved compared to the simple designs. The utilization of chaotic advection and converging–diverging mixing mechanisms enhances turbulent flow formation through eddies and vortices. These mixing effects were generated using serpentine structures, internal channel grooves and multichannel interlinking. Amongst the complex adsorbent microstructure designs developed and tested, the 2DH design consistently performed with the most favorable adsorption characteristics: long breakthrough time, high equilibrium loading, and a relatively short MTZ length. Results from both *n*‐butane breakthrough experiments conducted at constant flowrate and constant Reynolds number corroborate this finding. The 2DH design incorporates the benefit of having repeating crossing regions within the channels without reducing the surface contact between the carbon wall and the *n*‐butane. These benefits are also achieved without the trade‐off of having to significantly increase the tortuosity from the simple channel designs. SSG also demonstrated favorable adsorption characteristics in comparison to simple channel geometries and the complex counterparts. This geometry employed the use of serpentine channel structures and secondary flows through grooves in order to extend residence time within the structure through increasing the true path length of the channel, while introducing inherent mixing.

## 4 Conclusion

Through the use of SLA, a variety of adsorbent microstructures were made from a polyacrylate‐based resin, which was 3D printed and then successfully carbonized and activated to produce an adsorbent microstructure. The channel geometries were varied from straight‐through designs with conventional entrances, to complex micromixer designs that employed the use of serpentine and multichannel interlinking in order to increase the breakthrough time for *n*‐butane, shorten mass transfer zone length and maximize the equilibrium loading of the microstructures. Doing so developed effects such as chaotic advection and converging‐diverging networks within the microstructures increasing mixing and turbulence within the model. Breakthrough testing with 1000 ppm *n*‐butane at constant Reynolds number and at constant flow rate of 1 L min^−1^ revealed improved breakthrough times and higher equilibrium loadings in all adsorbent microstructure geometries compared to a granular carbon packed bed, which is typical in industry. For *n*‐butane breakthroughs at constant Reynolds number the breakthrough time and equilibrium loading were enhanced by up to 190% and 70% respectively. Compared to simple designs, the complex adsorbent microstructure designs gave rise to increased breakthrough time and equilibrium loading with reduced MTZ length demonstrating the benefit of novel geometries within the microstructures. Adsorbent microstructures also demonstrated ease of regeneration under vacuum at 150°C with regenerated samples maintaining consistent physical adsorption dynamics. While achieving enhanced *n*‐butane breakthrough qualities, the microstructures also maintained a significantly lower pressure drop than that of the GAC‐packed bed, in some cases up to 95% less. Out of the complex geometries tested, the 2D Helix and the Serpentine Spiral Grooves geometries were found to consistently provide the most favorable physical adsorption dynamics for the performance indicators examined.

## 5 Experimental Section

**Figure 9:**
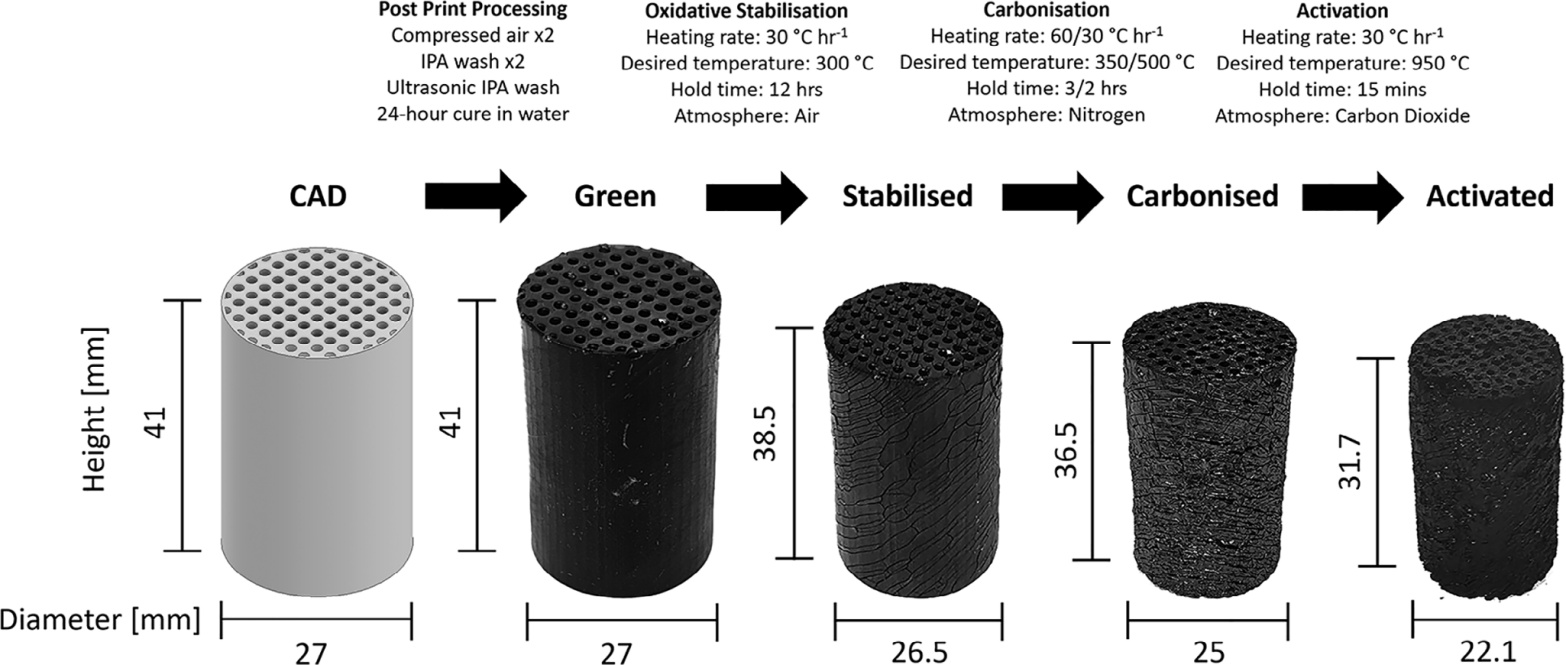
Example process demonstration of adsorbent activated carbon microstructure protocol for the circular geometry.

The design, production and testing of the activated carbon microstructures are summarized in Figure 3 which details each stage in the production process of the microstructures with visual changes. The CAD stage has been discussed previously. The processing stages described in **Figure** **9** are detailed within this section.

### Print and Post‐Print Processing of 3D Printed Microstructures

The simple and complex microstructures were printed using *Kudo3D's UHR Green* polyacrylate‐based resin. The printer used was a *Titan 2 HR* by *Kudo3D* with the print parameters found in Section S2 (Supporting Information).

Post‐processing of the freshly printed “green” microstructures involved blowing out excess resin trapped within the structure with compressed air. The microstructures were then washed in two separate baths of isopropyl alcohol for 5 min each and then processed through an ultrasonic cleaner with isopropyl alcohol for a further 5 min to remove any residual uncured resin within the structure. Remaining isopropanol was flushed out with compressed air and the adsorbent microstructures were cured in water and exposed to visible light for 24 h.

### Oxidative Stabilization, Carbonization, and Activation of 3D Printed Microstructures

Microstructures were physically activated in a tubular furnace through a three‐stage heating protocol (Figure 9). The initial step, oxidative stabilization, was conducted in air to a temperature of 300 °C with a heating rate of 30 °C h^−1^ to introduce more oxygen complexes on to the surface of the structure. Subsequently, the microstructures were carbonized in nitrogen at 500 °C. This occurred at a heating rate of 60 °C h^−1^ up to 350 °C, which was held for 3 h, and then heated to 500 °C at a rate of 30 °C h^−1^ and held for 4 h. Activation was conducted immediately after carbonization, with a rate of 30 °C h^−1^ for 4 h to 950 °C, followed by a 15‐min hold at 950 °C. The activation agent used was carbon dioxide. All of the heating protocol stages were performed at ambient pressure.

### Granular Activated Carbon (GAC) Packed Bed

GAC in a packed bed was used to act as a benchmark comparison for the AC microstructures. The GAC used was a Universal First Responder (UFR) GAC manufactured by Calgon Carbon. The sample was sieved to mesh size 32 (particle size range 0.5–1 mm), had a sphericity of 0.69 and a bed voidage of 0.37. This sample of UFR carbon was chosen as a representative carbon frequently used in industry. It was acknowledged that the UFR carbon, as received, was an impregnated form as its purpose is to tackle a wide range of chemicals. As a result, its equilibrium loading for physisorbed chemicals was compromised. The mass of GAC used was 4.05 g. This was used for both the *n*‐butane breakthrough and pressure drop testing.

### *n*‐Butane Breakthrough Testing

**Figure 10:**
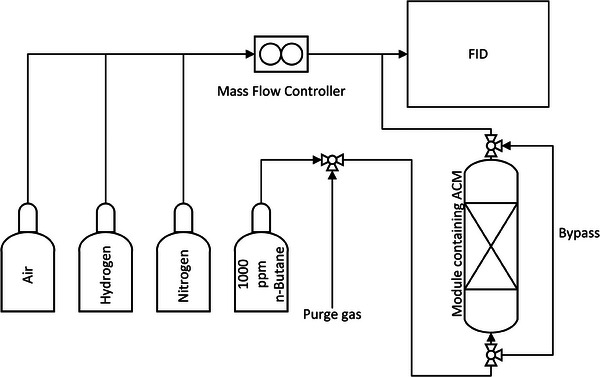
Schematic showing the *n*‐butane adsorption apparatus.

The physical adsorption properties for each microstructure design were evaluated by performing breakthrough experiments with *n*‐butane using the adsorption breakthrough apparatus illustrated in **Figure** **10**. This apparatus consists of a feed gas flow system, a stainless‐steel tubular module, and a flame ionization detector (*Teledyne Analytical Instrument 4030 series FID*). The apparatus was housed in a temperature‐controlled cabinet to maintain constant temperature conditions at a setpoint of 25 °C. For details on how breakthrough data was interpreted, see Section S3 (Supporting Information).

The AC material (microstructure or packed bed of granular activated carbon GAC) was secured in the center of the module between two meshes to ensure that the granular packed bed and microstructure was compacted with no gas flow bypassing the structure. 1000 ppm *n*‐butane in nitrogen was then fed into the module using a mass flow controller at the set flowrate. Hydrogen at 3.5 psig and nitrogen at 9 psig were used as the fuel and carrier gas for the FID, respectively.

The breakthrough experiments were evaluated at both constant Reynolds number (*Re* = 83) and at a constant 1 L min^−1^ flow rate. According to Equation (6), for the initial Reynolds number to remain constant the flow rate should be adjusted with the change of hydraulic diameter of the inlets (see Section S1, Supporting Information).

(6) $$Re=\frac{\rho v_{ave}d}{\mu }$$

where ρ is the gas density, μ is the dynamic viscosity, v_*ave*_ is the average velocity in the channels. To evaluate the hydraulic diameter of the activated microstructures (see Figure 9), microscopic images and ImageJ were used to estimate measurements of the channel inlets. For each of the novel designs (see Figure 2), at least three samples were printed and tested to examine the reproducibility of the printing, heating protocol, and adsorption results.

To assess the adsorbent microstructures potential for regeneration, the repeated samples were subjected to a regeneration cycle at 150 °C for 2 h under vacuum.

### Pressure Drop Testing

**Figure 11:**
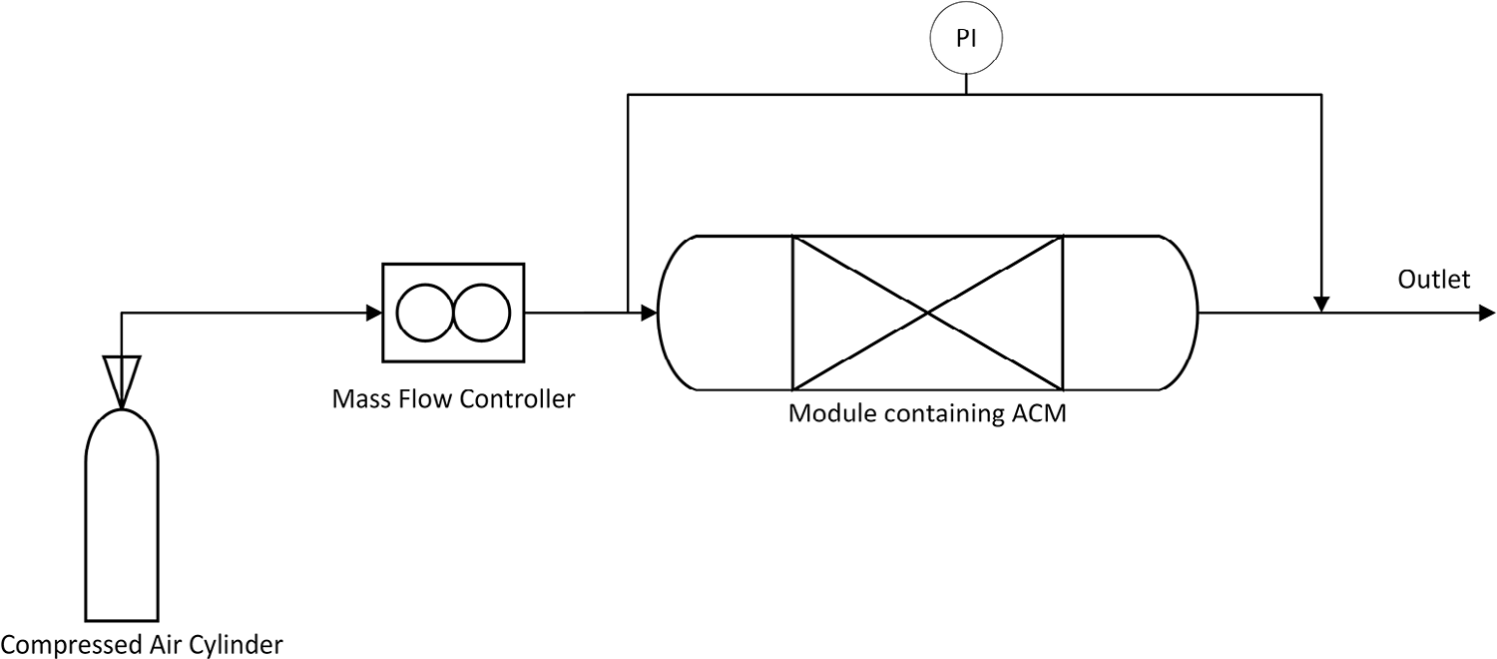
Schematic diagram showing pressure drop apparatus.

The resistance to flow of each channel design was characterized through pressure drop testing of the adsorbent microstructures and compared to a GAC‐packed bed. The AC microstructures were secured into the module using polystyrene to ensure that gas went through the structure rather than around. The GAC‐packed bed was compacted between two mesh gauzes. As seen in **Figure** **11**, a pressure transducer was used to measure the pressure differential between the inlet and outlet of the module at varying flowrates (0.2 to 2 L min^−1^) of compressed air.

Separate experiments were carried out to determine the pressure losses due to pipe friction, tube fittings, and roughness within the module not containing the microstructure. Therefore, when the microstructure was secured into the module, pressure losses attributed to the microstructure and not the module, could be ascertained.

### Characterization and Isotherm Measurement

Scanning electron microscopy (SEM) images were taken using a *JEOL JSM‐7900F FESEM*. Prior to imaging the samples were cut to size and coated with gold via sputter deposition. Computed tomography (CT) scans were carried out using a *Nikon XT H 225*, the samples were placed on a porous foam holder enabling ease of penetration for the X‐ray beams. Each sample was then placed on a manipulator plate, centered and then finally imaged. These scans were then compiled using *Avizo 9.2*.

Nitrogen isotherms at 77 K were carried out using a *Quantachrome Autosorb‐iQ* to determine the surface area and pore size distribution of the carbon samples. The QSDFT method for slit/cylindrical shaped carbon pores was used to analyze the pore size and the BET method to determine the surface area. The samples used in these experiments were different to those used in the *n*‐butane breakthrough and pressure drop.

### Compressive Strength Testing

The microstructures, TES, SSG, and 2DH, were tested using an *Instron Model 3369*. The microstructures were secured and centred beneath the contact block and then compressed at a rate of 2 mm min^−1^ with a load cell weight of 1kN. The compressive rate was chosen according to *ASTM C695‐15*.^[^ ^48^ ^]^

## Conflict of Interest

The authors declare no conflict of interest.

## Supporting information

Supporting Information

## Data Availability

The data that support the findings of this study are available from the corresponding author upon reasonable request.
