# Supplementary material for: A New Generation of Activated Carbon Adsorbent Microstructures
Source: Adv Sci (Weinh). 2024 Sep 6;11(42):2406551. doi: 10.1002/advs.202406551 (PMC11558120; doi:10.1002/advs.202406551)
Supplement: Supplementary file 1 — Supporting Information [file ADVS-11-2406551-s001.pdf]

ADVANCED SCIENCE
Open Access

Supporting Information

for *Adv. Sci.*, DOI 10.1002/advs.202406551

A New Generation of Activated Carbon Adsorbent Microstructures

Ethan Grigor, Joseph Carver*, Edric Bulan, Stuart Scott, YM John Chew and Semali Perera*

Supplementary Information

S.1 Summary of AC Microstrucutres Parameters

Table S1. The parameters found in the below table were measured post-activation of the microstructures tested at constant *Re* (83). This was used to establish testing conditions and calculate breakthrough properties.

	Channel designs	Height, H [mm]	Diameter, D [mm]	Channel Diameter, D_{CH} [mm]	Wall Thickness, t_w [mm]	True Path Length, L_{true} [mm]	CPI [-]
Simple	Circular (CIR)	29.82	18.61	1.08	0.71	29.82	201
	Square (SQR)	29.29	17.93	1.08	0.72	29.29	201
	Tessellated (TES)	31.95	19.65	1.04	0.66	31.95	223
Complex	Rifled Spiral (RS)	29.80	17.90	1.08	0.64	29.80	218
	Simple Serpentine (SS)	29.45	18.04	1.09	0.65	33.75	213
	2-Dimensional Helix (2DH)	29.21	18.00	1.10	0.66	33.48	208
	Serpentine Spiral Groove (SSG)	29.78	17.54	1.10	0.66	34.13	208
	3-Dimensional Helix (3DH)	29.43	18.53	1.15	1.63	37.63	83

Table S2. The parameters found in the below table were measured post-activation of the microstructures tested at constant flowrate 1 L min^{-1}. This was used to establish testing conditions and calculate breakthrough properties.

	Channel designs	Height, H [mm]	Diameter, D [mm]	Channel Diameter, D_{CH} [mm]	Wall Thickness, t_w [mm]	True Path Length, L_{true} [mm]	CPI [-]
Simple	Circular (CIR)	28.67	18.20	1.08	0.67	28.67	211
	Square (SQR)	29.68	18.06	1.10	0.73	29.68	193
	Tessellated (TES)	30.31	18.76	1.04	0.69	30.31	216
Complex	Rifled Spiral (RS)	29.74	17.57	1.02	0.62	29.74	240
	Simple Serpentine (SS)	29.45	18.04	1.09	0.65	33.75	213
	2-Dimensional Helix (2DH)	28.55	18.48	1.03	0.62	34.13	237
	Serpentine Spiral Groove (SSG)	29.78	17.54	1.10	0.66	34.13	208
	3-Dimensional Helix (3DH)	28.71	18.29	1.19	1.63	36.68	83

Degree of activation is defined as the mass of carbon remaining after activation and was calculated using Equation S.1.

$$DoA\% = 100\frac{M_{ac}}{M_{carb}} \tag{S.1}$$

where DoA is the degree of activation, M_{ac} is the mass of material after activation and M_{carb} is the mass of material after carbonization.

S.2 Print Parameters Used for Titan 2 HR

Table S3. All microstructures were printed using the below parameters.

From Layer	To Layer	Exp. Time [s]	Lift Height [mm]	Lift Speed	Down Speed
1	1	25	5	5	150
2	9	12	5	5	150
10	80	4	2.5	2.5	150
81	120	4	3	3	150
121	200	4	3	3	150
201	400	4	2.5	2.5	150
401	700	3	2	2	150
701	1000	2	2	2	150
1001	2116	2	2	2	150

S.3 n-butane Breakthrough Analysis

All ACs tested with n-butane generated a breakthrough curve that was normalised by weight (Figure S1). From this breakthrough profile, breakthrough time, t_b, equilibrium loading, q_e, and mass transfer length, *MTZ Length*, can be calculated using the following equations, S.2 and S.3.

$$q_e = \frac{Q_b M_w C_0 t_t - Q_b M_w \int_0^{t_t} C_t dt}{m_c} \tag{S.2}$$

$$MTZ = L\frac{t_{eq} - t_b}{t_{eq}} \tag{S.3}$$

Where Q_b is the flowrate of butane, M_w is the molecular mass of butane, C_0 is the initial concentration of butane, t_t is the total testing time, C_t is the concentration of butane at time t, m_c is the mass of carbon, L is the length of the AC microstructure and t_{eq} is the time taken to reach 90% of the inlet concentration in the FID.

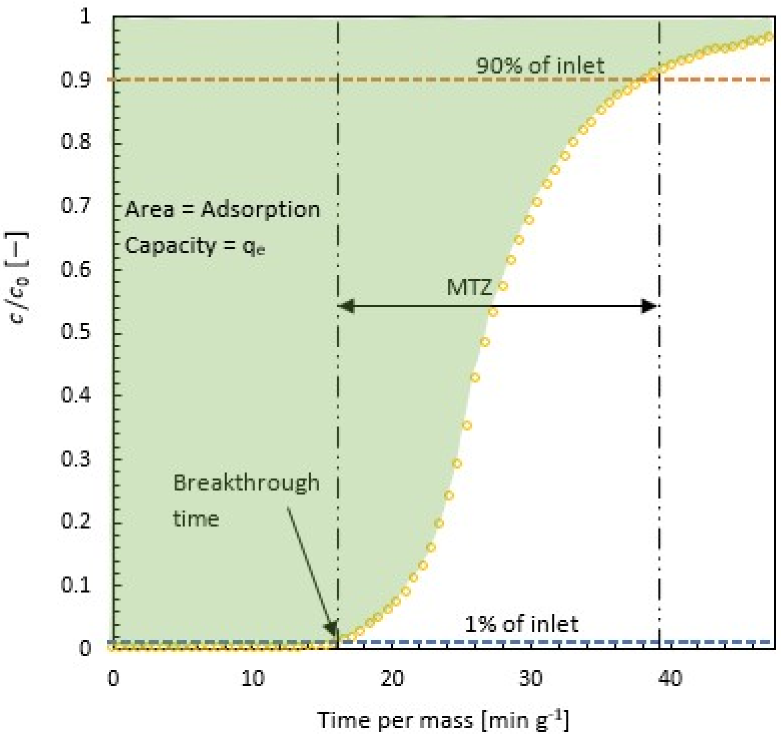

Figure S1. Example physisorption breakthrough curve showing the breakthrough time (1%), the mass transfer zone length (1% to 90%) and the green area which represents the equilibrium capacity.

S.4 SEM micrographs for simple and complex channel geometries

SEM micrographs can be seen in Figure S2 with their corresponding viewpoints. Highlighted sections show the channel pathways.

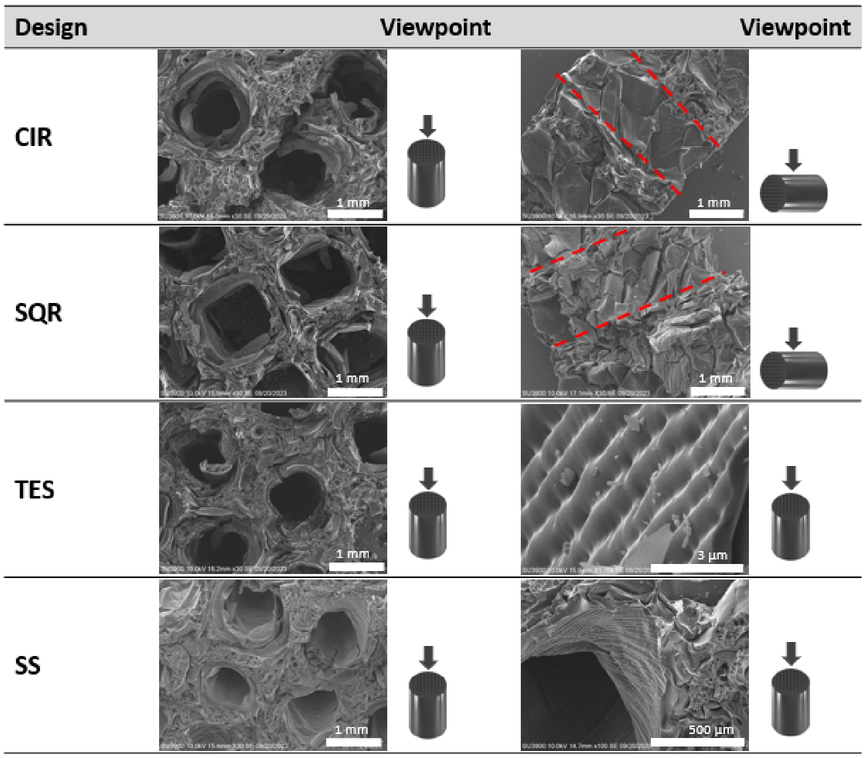

Figure S2. SEM images from the top and interior of the channels and cross-sectional view.

S.5 Nitrogen isotherms at 77 K for AC microstructures and GAC.

The isotherms in Figure S3 were used to calculate the BET surface area of the ACs and the pore size distribution using the QSDFT method.

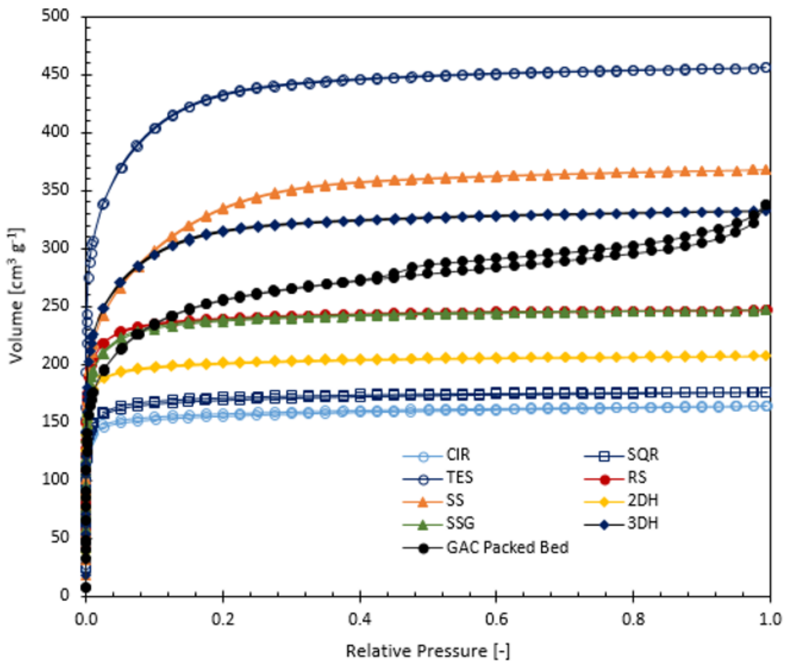

Figure S3. Nitrogen isotherms for activated microstructures and GAC taken at 77 K.

S.6 Reproducibility of microstructures demonstrated by *n*-butane breakthrough profiles.

Figure S4 shows breakthrough profiles where different 2DH samples that were tested at constant *Re*= 83, all yielding similar shaped curves. A table of their corresponding adsorption performances (t_b, q_e and *MTZ*).

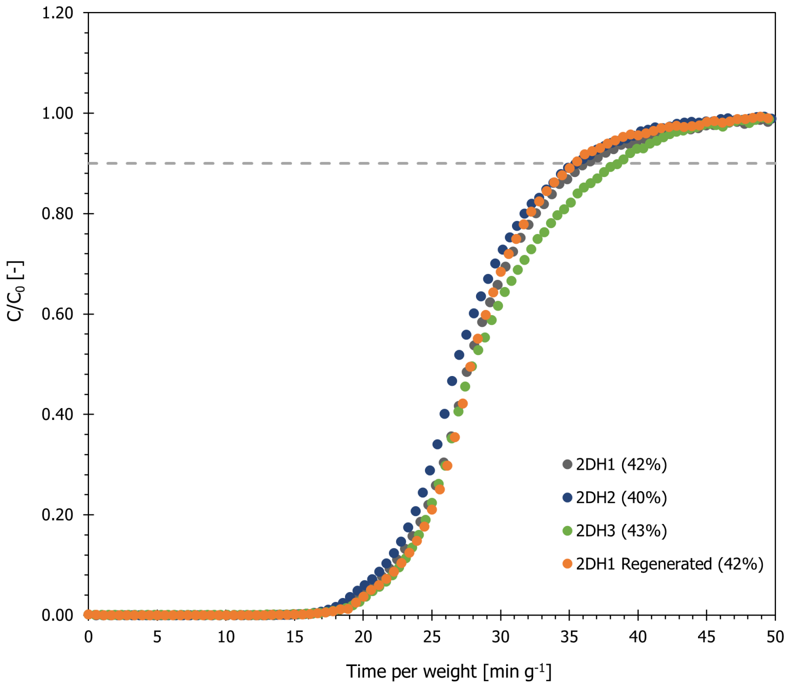

Figure S4. Different 2DH microstructure samples *n*-butane breakthrough curves.

Table S4. Summary of adsorption performance.

Channel Designs	Flow Rate [L min^{-1}]	Degree of Activation [%]	Breakthrough Time t_b [min g^{-1}]	Equilibrium Loading, q_e [g g^{-1}]	MTZ Length [mm]
**2DH1**	1.08	42	17.7	0.082	15.0
**2DH1 Regen**	1.08	41	18.0	0.082	14.0
**2DH2**	1.08	39.8	17.2	0.083	15.2
**2DH3**	1.01	43	17.9	0.084	14.1
